# Supplementary material for: Proteomic Analysis of Alfalfa (Medicago sativa L.) Roots in Response to Rhizobium Nodulation and Salt Stress
Source: Genes (Basel). 2022 Nov 2;13(11):2004. doi: 10.3390/genes13112004 (PMC9690670; doi:10.3390/genes13112004)
Supplement: Supplementary file 1 [file genes-13-02004-s001.zip › supplementary figures.pdf]

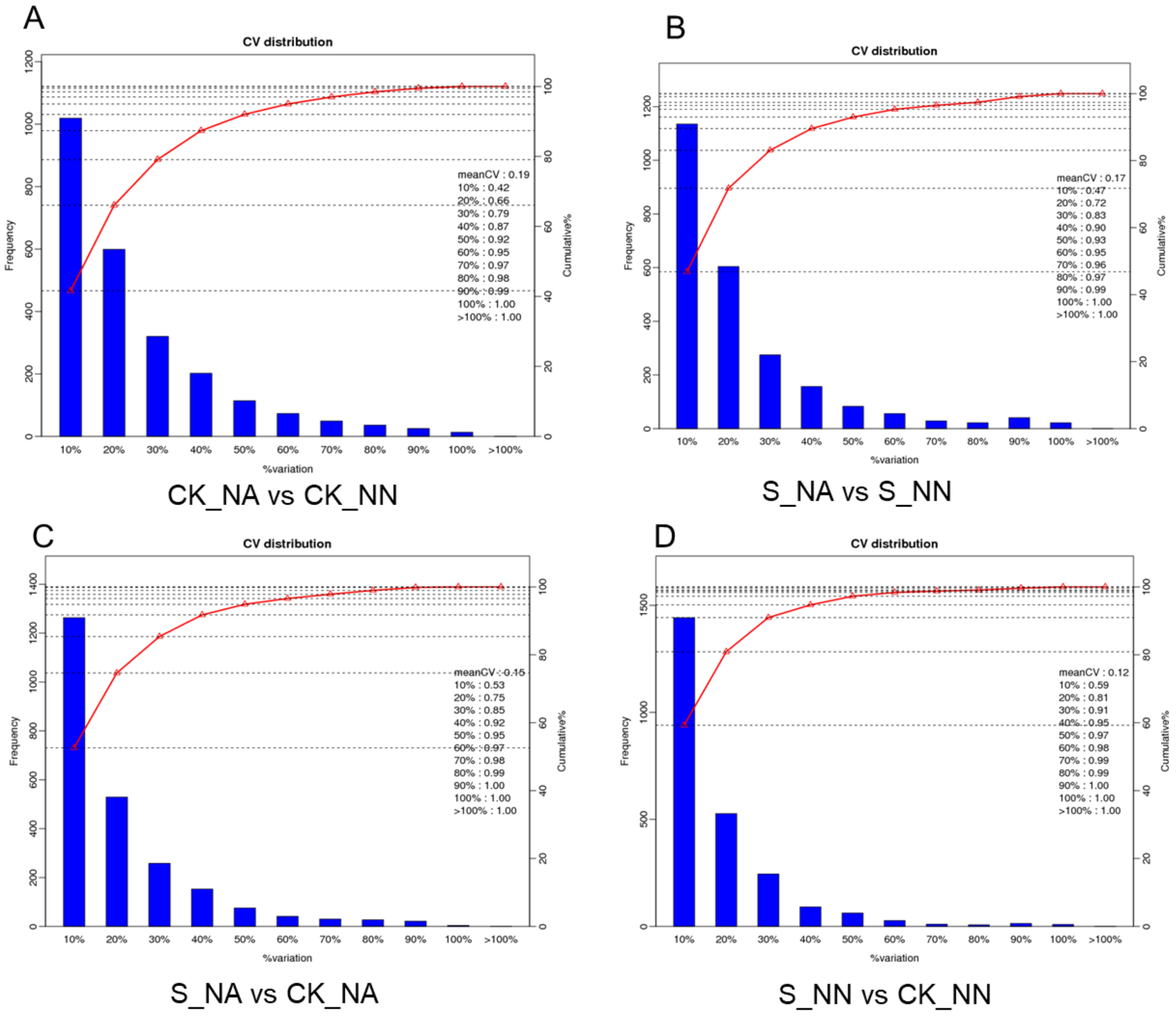

Figure S1: Repeatability analysis based on the coefficient of variation (CV) for comparing CK_NA and CK_NN (A); S_NA and S_NN (B); S_NA and CK_NA (C); S_NN and CK_NN (D). The left vertical axis shows the frequency of the quantitative proteins, and the right vertical axis represents the cumulative percentage of proteins. S: alfalfa under salt stress; CK: alfalfa under normal conditions; NA: alfalfa plants with active nodules; NN: alfalfa plants without nodules

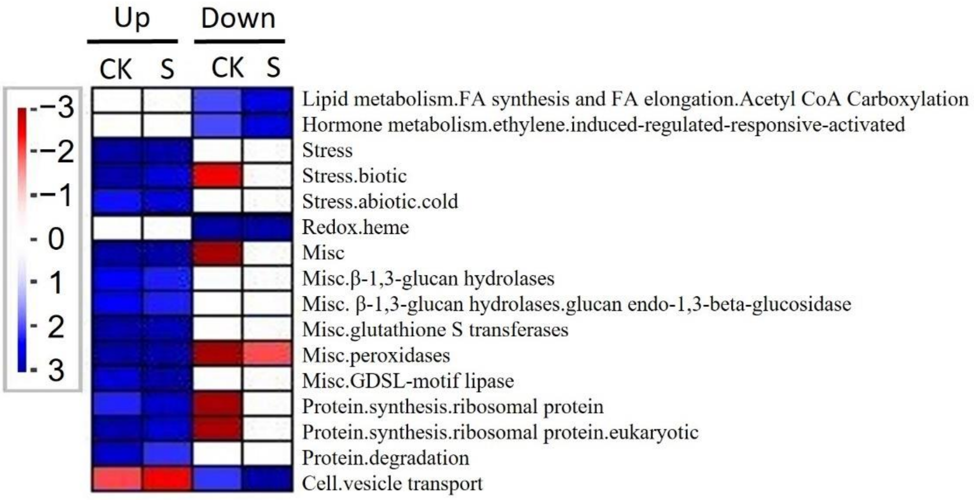

Figure S2: The common pathways shared by alfalfa roots under normal growth conditions and salt stress conditions when comparing the NA and NN plants. Functional categories enrichment was made with Fisher's exact. blue color: pathways were significantly over-represented; red: pathways were significantly under-represented. NA: alfalfa plants with active nodules; NN: alfalfa plants without nodules
